# Physical Activity and Its Effects on Executive Functions and Brain Outcomes in Children: A Narrative Review

**DOI:** 10.3390/brainsci15111238

**Published:** 2025-11-18

**Authors:** Eduardo Guzmán-Muñoz, Yeny Concha-Cisternas, Emilio Jofré-Saldía, Antonio Castillo-Paredes, Iván Molina-Márquez, Rodrigo Yáñez-Sepúlveda

**Affiliations:** 1 Escuela de Kinesiología, Facultad de Salud, Universidad Santo Tomás, Talca 3460000, Chile; 2 Pedagogía en Educaciόn Física, Facultad de Educaciόn, Universidad Autόnoma de Chile, Talca 3460000, Chile; 3 Vicerrectoría de Investigaciόn e Innovaciόn, Universidad Arturo Prat, Iquique 1100000, Chile; 4 Escuela de Ciencias de la Actividad Física, Facultad de Ciencias de la Rehabilitación y Calidad de Vida, Universidad San Sebastián, Santiago 8370040, Chile; emilio.jofre@uss.cl; 5 Grupo AFySE, Investigación en Actividad Física y Salud Escolar, Escuela de Pedagogía en Educación Física, Facultad de Educación, Universidad de Las Américas, Santiago 8370040, Chile; acastillop85@gmail.com; 6 Escuela de Educación Física, Facultad de Educación, Universidad Adventista de Chile, Chillán 3780000, Chile; ivanmolina@unach.cl; 7 Programa Doctorado en Ciencias de la Actividad Física, Universidad Católica del Maule, Talca 3460000, Chile; 8 Faculty Education and Social Sciences, Universidad Andrés Bello, Viña del Mar 2520000, Chile; rodrigo.yanez.s@unab.cl; 9 School of Medicine, Universidad Espíritu Santo, Samborondón 092301, Ecuador

**Keywords:** exercise, inhibitory control, working memory, cognitive flexibility, neuroplasticity, children, attention-deficit/hyperactivity disorder

## Abstract

**Background/Objectives:** Executive functions—including inhibitory control, working memory, and cognitive flexibility—are fundamental for children’s learning and development. Physical activity is recognized as a key factor that enhances these functions through neurobiological and structural brain adaptations. This narrative review aims to synthesize current evidence on the relationship between physical activity, executive functions, and brain outcomes in children. **Methods:** A narrative review was conducted using systematic evidence search across PubMed, Scopus, and Web of Science until August 2025. Search terms encompassed physical activity, executive functions, and brain mechanisms. Eligible studies included randomized controlled trials, longitudinal and cross-sectional studies, systematic reviews, and meta-analyses that examined executive function domains and brain-related outcomes in children, with or without neurodevelopmental disorders. **Results:** The evidence reviewed highlights that acute physical activity improves inhibitory control, working memory, and cognitive flexibility, primarily through enhanced neurotransmission and cerebral oxygenation. Chronic interventions promote structural and functional brain adaptations, including improved white matter integrity and increased network efficiency. Benefits are observed in both neurotypical children and those with ADHD, with inhibitory control emerging as the most responsive domain. However, findings are moderated by intervention type, intensity, and duration, with heterogeneity across protocols. **Conclusions:** Physical activity is a promising strategy to support the development of executive and brain functions in childhood, with implications for education and clinical practice. Despite consistent short- and long-term benefits, further research is required to establish optimal prescriptions and evaluate sustained real-world impacts, particularly in children with neurodevelopmental disorders.

## 1. Introduction

Childhood is a critical stage for the development of executive functions, which include cognitive skills such as inhibitory control, working memory, and cognitive flexibility [1,2]. These abilities play a fundamental role in supporting learning processes and regulating behavior, making them essential for children’s academic performance and daily life [1,3]. This period is also marked by heightened neuroplasticity, with rapid structural and functional brain changes [4]. These processes are strongly shaped by the surrounding environment, and among the factors that influence them, physical activity has emerged as a powerful stimulus for both executive functions and overall brain health [5,6].

Globally, 81.0% of adolescents aged 11–17 years were insufficiently physically active in 2016, including 77.6% of boys and 84.7% of girls, according to a pooled analysis of 1.6 million students from 146 countries, in which insufficient physical activity was defined as not meeting the WHO recommendation of at least 60 min of moderate-to-vigorous physical activity per day [7]. Such widespread inactivity raises serious concerns not only for children’s physical health but also for their cognitive development and overall brain function.

Growing evidence shows that regular physical activity supports brain development and cognitive performance in children through multiple biological mechanisms. Exercise increases the release of neurotrophic factors such as brain-derived neurotrophic factor (BDNF), which enhance neuronal survival, synaptic plasticity, and neuroadaptation [8]. It also acutely increases cerebral blood flow, improving the delivery of oxygen and nutrients to the brain [9]. Over time, these effects contribute to structural and functional adaptations, including greater white-matter integrity and stimulated neurogenesis—both key processes for executive functioning [10]. Even a single exercise session can produce short-term gains in inhibitory control and attention, likely mediated by transient increases in BDNF and arousal [8,11]. In the long term, consistent participation in physical activity and sports has been associated with superior executive function and self-regulation in childhood [12]. Overall, physical activity appears to act both as an acute modulator and a long-term driver of neurocognitive development.

However, several critical gaps remain in the literature. It is still unclear which specific types or intensities of physical activity yield optimal benefits for children’s executive functions [13]. Most available studies have been short-term interventions, providing limited evidence on the long-term developmental effects [14]. Moreover, the neurobiological mechanisms through which exercise influences executive and brain functions in children—particularly in those with neurodevelopmental disorders—are not yet fully understood [13].

Notably, there is a paucity of research directly comparing neurotypical children with those who have neurodevelopmental disorders, particularly attention-deficit/hyperactivity disorder (ADHD). Only a limited number of studies have examined the effects of exercise on executive functions in this population, leaving this area largely unexplored [15]. Addressing these gaps is especially important and timely, given the alarming global prevalence of physical inactivity among children and the growing interest in non-pharmacological strategies to support healthy brain development [16]. Therefore, this narrative review aims to synthesize current evidence on how physical activity influences executive and brain functions in children, encompassing both neurotypical populations and those with neurodevelopmental disorders. By identifying key gaps and implications, the review seeks to clarify the current state of knowledge and inform future research and practice.

## 2. Materials and Methods

This study was designed as a narrative review with a structured and comprehensive literature search. Searches were conducted in PubMed, Scopus, and Web of Science from inception to 31 August 2025. The search strategy combined terms related to physical activity, executive functions, and brain mechanisms in children. Keywords included: *“physical activity”*, *“exercise”*, *“sport”*, *“executive function”*, *“executive control”*, *“cognitive control”*, *“inhibitory control”*, *“working memory”*, *“cognitive flexibility”*, *“self-regulation”*, *“attention”*, *“brain function”*, *“cognition”*, *“neurocognition”*, *“neuroplasticity”*, *“synaptic plasticity”*, *“functional connectivity”*, and *“prefrontal cortex”*. Boolean operators (AND/OR) were applied to ensure sensitivity and specificity. Additional records were identified by manually screening the reference lists of eligible articles and related reviews. No date or language restrictions were applied.

Two independent reviewers conducted the screening process, including duplicate removal, title and abstract screening, and full-text assessment. Discrepancies were resolved by discussion and consensus. Rayyan software (http://rayyan.qcri.org, accessed on 1 August 2025) was used to support duplicate removal and study selection.

The inclusion criteria comprised:

- Population: children and adolescents (neurotypical or with neurodevelopmental conditions).
- Intervention/exposure: acute or chronic, structured or unstructured physical activity or exercise.
- Outcomes: executive function domains (inhibitory control, working memory, cognitive flexibility, self-regulation, attention) and/or measures of brain function and neuroplasticity (cognition, connectivity, BDNF, cerebral blood flow, neuroimaging outcomes).
- Study designs: randomized controlled trials (RCTs), longitudinal and cross-sectional studies, systematic reviews, and meta-analyses.

Eligibility was assessed according to the alignment of each study with the predefined thematic areas and the transparency of the reported findings. Priority was given to original research articles that directly examined the concepts of interest, as well as to systematic reviews that synthesized the available evidence. When two publications presented overlapping results, preference was given to the most recent and comprehensive study. The exclusion criteria were: commentaries, letters to the editor, conference abstracts, study protocols, and trial registrations.

All relevant data were extracted and synthesized qualitatively following a framework established a priori by the research team. The analysis was structured into five thematic areas (inhibitory control, working memory, cognitive flexibility, brain function, and neurodevelopmental disorders), reflecting the most commonly studied domains in the literature.

The organization of this review follows the classical theoretical model of executive functions employed in the literature [1,3,15,17], which distinguishes three core domains: inhibitory control, working memory, and cognitive flexibility.

## 3. Results

### 3.1. Physical Activity and Executive Functions: Neurobiological Pathways

The effect of physical activity on executive functions is mediated by a set of interconnected neurobiological pathways that operate both acutely and chronically. These mechanisms represent a common framework that underpins improvements in inhibitory control, working memory, and cognitive flexibility, with domain-specific nuances emerging depending on the task demands and developmental stage.

At the neurochemical level, acute exercise elevates catecholaminergic neurotransmission—particularly dopamine and norepinephrine—through the locus coeruleus–norepinephrine (LC-NE) system. This increase enhances cortical arousal and optimizes the signal-to-noise ratio in task-relevant circuits [11,17]. In parallel, physical activity upregulates neurotrophic factors such as BDNF and insulin-like growth factor 1 (IGF-1), which promote synaptic plasticity, long-term potentiation, and hippocampal neurogenesis [8,17,18].

At the cerebrovascular level, exercise acutely augments cerebral blood flow and prefrontal oxygenation, facilitating efficient resource allocation during executive tasks [9,19]. With repeated practice, chronic exercise induces structural adaptations, including improved white matter integrity (e.g., in the corpus callosum) and gray matter refinements in the prefrontal and parietal cortices [18,20]. These adaptations support the long-term consolidation of executive improvements.

At the network level, functional neuroimaging and electrophysiological studies converge to show that physical activity strengthens large-scale networks that subserve executive control. Acute bouts increase activation and connectivity in the dorsolateral prefrontal cortex, anterior cingulate cortex, and parietal regions [21,22,23], while modulating electrophysiological markers such as larger P3 amplitudes, reduced N2 latencies, and enhanced contingent negative variation [24,25,26,27,28]. Over time, chronic engagement in exercise promotes greater efficiency within frontoparietal and cingulo-opercular networks, which are crucial for flexible, goal-directed behavior [20,29,30,31].

Taken together, these mechanisms highlight that physical activity exerts its cognitive benefits through convergent pathways—catecholaminergic modulation, neurotrophin release, hemodynamic facilitation, and network efficiency—that operate synergistically to enhance executive functions. While these processes are largely shared across domains, each executive subcomponent (inhibitory control, working memory, and cognitive flexibility) displays distinct sensitivity to particular markers (e.g., N2/ERN for inhibition, hippocampal-prefrontal coupling for working memory, or switch-cost reduction for flexibility) [17,22,24,26,32]. This integrative framework emphasizes that the executive gains observed in children are not isolated phenomena but rather the result of systemic neurobiological adaptations elicited by regular physical activity.

### 3.2. Physical Activity and Inhibitory Control

Inhibitory control—defined as the capacity to suppress prepotent or irrelevant responses in favor of goal-directed behavior—is widely recognized as a core component of executive functioning in childhood [33]. It enables children to withhold impulsive actions, resist distractions, and select task-appropriate responses, thereby supporting classroom conduct, learning readiness, and social-emotional regulation [1]. Developmentally, inhibitory control follows the protracted maturation of fronto-parietal networks, particularly the prefrontal cortex, which continues to refine synaptic organization, myelination, and functional connectivity throughout childhood and adolescence [34]. Because these neural systems are still under construction during the early school years, this period is especially sensitive to environmental inputs—among them, physical activity—which can shape the efficiency and resilience of the circuits that subserve self-regulation [35]. Stronger inhibitory control in childhood has been associated with better academic achievement, more adaptive classroom behavior, and improved socioemotional outcomes over time; conversely, weaknesses in this domain are linked to distractibility, impulsivity, and difficulties sustaining goal-directed attention [3].

The effects of acute exercise on inhibitory control are among the most consistently observed in neurotypical pediatric samples. Following a single bout of moderate-to-vigorous activity (≈15–30 min of aerobic exercise or cognitively engaging active play), children typically show faster responses and/or reduced interference on canonical inhibition tasks (Stroop, Flanker, Go/No-Go), with accuracy maintained or slightly improved. Meta-analytic evidence in youth indicates a small but consistent acute benefit on inhibitory outcomes [36]. Classroom and laboratory experiments in school-age children—including cluster-randomized or counterbalanced designs—show that ~20 min of moderate physical activity is often sufficient to improve performance on interference-laden inhibitory tasks, with effects most evident when testing occurs shortly after exercise and, in classroom settings, tending to dissipate within ~30–40 min [24,37]. Protocols that embed cognitive demands (rules, rapid switching, decision-making) can elicit acute improvements; emerging evidence suggests this may amplify post-exercise gains, although direct head-to-head comparisons with duration- and intensity-matched monotonous aerobic exercise remain limited [24,36,37]. Across studies, the acute profile reflects greater processing efficiency—shorter reaction times, smaller interference costs, and in some cases fewer commission errors—without a speed–accuracy trade-off [24,36,37]. Regarding dose, very brief or very light activity frequently shows trivial effects, whereas the most consistent benefits are observed after moderate-to-vigorous sessions of ~15–30 min. Evidence at very vigorous intensities is mixed: some studies have reported transient declines in inhibitory performance immediately after exhaustive exercise—such as slower reaction times or reduced accuracy in Stroop and Flanker tasks—likely due to fatigue and excessive arousal, while others observed recovery and even improvement after short rest intervals [24,36,37,38,39].

The chronic effects of regular physical activity on inhibitory control present a more complex profile than acute responses, with emerging evidence supporting modest but meaningful improvements following sustained exercise interventions. Meta-analytic evidence from systematic reviews targeting healthy children and adolescents indicates that chronic exercise interventions produce small but statistically significant effects on inhibitory control accuracy [36,40]. Dose appears relevant: multi-week programs (typically ≥ 6–12 weeks) delivering moderate-to-vigorous sessions of ~20–40 min on most days of the week are frequently associated with small but significant improvements in inhibitory control; however, available research, including meta-analyses and individual trials, has not consistently identified a single optimal duration or frequency of intervention to maximize inhibitory control improvements [36,40,41]. The FITKids randomized controlled trial—9 months of daily after-school moderate-to-vigorous physical activity in preadolescent children—yielded significant improvements in inhibitory control, evidenced by better performance on a modified Flanker task and increased P3 amplitude during inhibition demands; moreover, higher program attendance was associated with larger gains in neuroelectric indices on the inhibition task [25,26]. Emerging evidence suggests dose-dependent effects, as demonstrated in adolescent samples where high-dose exercise protocols (two 30 min sessions daily) yielded significantly greater improvements in interference scores compared to low-dose interventions, with changes in physical activity levels correlating with inhibitory control enhancements [41]. In real-world settings, moderate-intensity programs delivering 30–50 min sessions at least three times per week for ≥17 weeks are consistently associated with improvements in inhibitory control. Among long-term approaches, interventions that incorporate open, sequential, or open-sequential motor skills have been most frequently linked to larger gains in inhibitory control [42]. However, direct comparisons between aerobic and coordinative training have yielded mixed results, with some randomized controlled trials finding no differential effects on inhibitory control measures despite improvements in fitness parameters [43]. Taken together, multi-week programs with regular moderate-to-vigorous sessions are associated with small but meaningful gains in inhibitory control; meta-analytic moderator analyses have not consistently identified a single optimal duration or frequency [36,40,42].

These improvements in inhibitory control likely reflect, at least in part, the acute upregulation of catecholamines—particularly dopamine and norepinephrine—via the LC–NE system, which enhances cortical arousal and optimizes the signal-to-noise ratio within prefrontal circuits. In the longer term, repeated engagement in physical activity promotes the release of neurotrophic factors such as BDNF and IGF-1, supporting long-term potentiation and structural refinement of the frontoparietal networks that sustain inhibitory processes, as detailed in Section 3.1.

### 3.3. Physical Activity and Working Memory

Working memory—defined as the cognitive system that temporarily holds and manipulates information necessary for complex mental operations—represents a fundamental component of executive functioning that supports academic learning, problem-solving, and goal-directed behavior in children [1,44]. According to Baddeley’s model, it includes the central executive, phonological loop, visuospatial sketchpad, and episodic buffer [44]. Developmentally, working memory capacity increases substantially during childhood, with improvements in processing speed, storage capacity, and manipulation efficiency paralleling the protracted maturation of frontoparietal networks and associated white matter connectivity [45]. Individual differences in working memory performance during childhood strongly predict academic achievement in reading, mathematics, and language comprehension, often serving as better predictors of educational success than traditional intelligence quotient measures [3,46]. Children with stronger working memory abilities demonstrate superior performance across multiple cognitive domains, including fluid intelligence, sustained attention, and inhibitory control, reflecting the system’s role as a cognitive foundation for complex learning processes [45].

Acute exercise confers reliable benefits to working memory in neurotypical children. Meta-analytic evidence from randomized trials indicates significant improvements in pediatric working memory after single sessions of physical activity, with a moderate effect size [36]. Acute benefits are most often detected when testing occurs shortly after moderate bouts, commonly around 20–30 min, though no single optimal bout length is established in pediatric samples [36]. The timing of acute exercise effects on working memory in neurotypical children appears task- and intensity-dependent. After moderate bouts, improvements are most often detected when testing occurs within ~0–20 min post-exercise; by contrast, following maximal exertion, immediate decrements can emerge on some tasks (e.g., verbal learning), with working-memory benefits appearing only after a recovery period (≈30–60 min) [47]. Cross-sectional evidence indicates that school-age girls who practice artistic gymnastics perform better on visuospatial working memory tasks than non-athletic peers, reinforcing the association between structured physical activity and specific cognitive domains [48]. However, this study does not address acute post-exercise timing, verbal vs. visuospatial contrasts, or causality. This behavioral pattern aligns with meta-analytic evidence showing small but significant improvements in visuospatial working memory in children following chronic, cognitively engaging, low-to-moderate intensity programs (sessions > 30 min, duration > 90 days); however, direct pediatric neuroimaging evidence remains limited, so mechanistic inferences about specific parietal (e.g., right-hemisphere) modulation should be considered preliminary [49]. Current pediatric evidence does not support a single ‘optimal’ dose: acute working-memory benefits are most consistently detected after ~20–30 min of moderate-to-high intensity sessions (mainly on response-time measures), whereas very light loads tend to be trivial and very vigorous bouts show mixed, task-dependent effects [36,50].

The chronic effects of structured physical activity on working memory in youth are modest but meaningful, primarily reflected in faster response times, with smaller and less consistent effects on accuracy [36]. Moderator analyses indicate that sessions ≤ 30 min delivered over <12 weeks are associated with larger gains in working-memory speed than longer sessions or extended programs; any attenuation with higher volumes may reflect fatigue-related effects and should be interpreted with caution [36]. Consistent with this pattern, a 12-week coordinative training program (~40 min, twice weekly) produced visuospatial working-memory gains on the Corsi block-tapping task versus an active control, although direct head-to-head comparisons with traditional aerobic training and effects on visuospatial attention remain limited [51]. Likewise, an 8-week school break–time program combining aerobic and coordinative exercise (20 min/day) in adolescents improved Sternberg working-memory response times without changing accuracy, accompanied by increased contingent negative variation over fronto-central regions—consistent with enhanced task preparation/proactive control [52]. Age-related moderation further indicates larger working-memory benefits in children aged 5–12 than in adolescents aged 12–18 [36]. Overall, programs up to ~12 weeks with cognitively engaging sessions of ≤30 min show the most consistent improvements in pediatric working memory—mainly as faster responses—whereas longer or more frequent volumes have not reliably produced additional gains [36,50].

The observed benefits in working memory following regular physical activity are likely underpinned by enhanced functional coupling between the hippocampus and prefrontal cortex, as well as increased cerebral oxygenation. These outcomes are facilitated by both aerobic and coordinative exercise modalities and can be mechanistically attributed to neurotrophic and cerebrovascular processes previously outlined in Section 3.1. Notably, upregulation of BDNF supports synaptic plasticity and structural refinement in white matter tracts, which optimize the updating and maintenance of information in working memory.

### 3.4. Physical Activity and Cognitive Flexibility

Cognitive flexibility—defined as the capacity to adapt one’s behavior in response to changes in the environment and shift flexibly between tasks, mental sets, or strategies—represents a fundamental component of executive functioning that enables adaptive regulation of thoughts and actions in dynamic contexts [1,53]. Also referred to as set-shifting or task-switching, cognitive flexibility encompasses the ability to switch attention between different tasks or goals, shift cognitive strategies when faced with new or unexpected situations, and overcome perseverative responding when previous approaches are no longer effective [54]. Developmentally, cognitive flexibility undergoes substantial improvement during childhood, with the most intensive development occurring between 7 and 12 years of age as prefrontal cortex and inferior parietal cortex maturation progresses alongside enhanced functional connectivity within frontoparietal networks [54,55]. Individual differences in cognitive flexibility during childhood strongly predict academic achievement, social competence, and behavioral adaptation, with stronger flexible thinking abilities associated with better problem-solving skills, creative thinking, and resilience to environmental changes [56]. Conversely, deficits in cognitive flexibility are linked to difficulties with transitions, perseverative behaviors, and challenges adapting to novel or changing situations, potentially impacting classroom performance and peer relationships [57].

The acute effects of physical activity on cognitive flexibility demonstrate consistent benefits across neurotypical pediatric populations. Meta-analytic evidence indicates that single bouts of exercise produce significant improvements in cognitive flexibility with small to moderate effect sizes, primarily reflected in faster response times and reduced switch costs on task-switching paradigms [36]. In individual experiments, a 20 min aerobic bout improved performance on a divergent-thinking flexibility task compared with a seated control condition, with heart-rate-variability changes consistent with heightened arousal [58]. These benefits typically emerge when assessment occurs soon after exercise; however, a single “optimal” bout duration has not been established in pediatric samples. Across pediatric and mixed-age syntheses, moderate intensities tend to be more reliable than very light or exhaustive bouts; activities that embed cognitive demands (e.g., rules, switching, rapid decision-making) may amplify benefits, although head-to-head pediatric comparisons with duration- and intensity-matched monotonic aerobic exercise remain limited [36,58].

The chronic effects of sustained physical activity interventions on cognitive flexibility present evidence supporting modest but meaningful improvements following long-term exercise programs. Meta-analytic findings from randomized controlled trials indicate that chronic physical activity interventions produce significant enhancements in cognitive flexibility performance with small to moderate effect sizes, demonstrating improvements primarily in response speed and set-shifting efficiency [36,40]. In a nine-month randomized after-school program with daily moderate-to-vigorous physical activity, preadolescent children showed greater gains in cognitive flexibility on a task-switching paradigm, with accompanying increases in P3 amplitude under flexibility demands and attendance correlating positively with performance change [26]. Across pediatric randomized trials and meta-analyses, multi-week, moderate-intensity programs show small but reliable gains in cognitive flexibility, typically as faster switching and reduced perseveration. A pragmatic prescription that yields the most consistent benefits is 8–12 weeks, 3–5 sessions/week, ~30–45 min per session, emphasizing cognitively engaging activities (open-skill games, coordinative drills, team sports, structured play with rules) [32,36,42,59]. Extending sessions well beyond ~45–50 min or pushing weekly volumes much higher has not consistently produced larger gains and may be vulnerable to fatigue-related attenuation; importantly, available research, including meta-analyses and individual trials, does not identify a single ‘optimal’ dose of physical activity for enhancing executive or brain functions in children [26,32,36]. Concerning exercise modality, coordinative programs that incorporate cognitive challenges appear to provide greater benefits for cognitive flexibility in neurotypical children than traditional aerobic exercise [13]. For example, studies with ~12-week coordinative training interventions have reported significant improvements in task-switching performance and greater ability to adapt to changing task demands, indicating enhanced cognitive flexibility [13]. Age-related moderator effects suggest that younger children (7–9 years) show greater responsiveness to cognitive flexibility interventions compared to older children (10–12 years), possibly reflecting critical periods of prefrontal cortex development and heightened neuroplasticity during earlier childhood [13]. School-based, cognitively engaging physical activity—especially open-skill/coordinative formats that require rule changes, strategic adjustments, and rapid decisions—delivered for >6 weeks with ≥3 sessions/week of ≥20 min at moderate intensity is associated with small but reliable improvements in cognitive flexibility; gains are most evident in multi-week trials that assess task-switching performance [60]. Taken together, multi-week, moderate-intensity programs that pair aerobic activity with cognitively engaging tasks yield small, reliable gains in cognitive flexibility—observed as faster task switching—while neurophysiological changes suggest (without definitively demonstrating) greater efficiency of control networks.

Gains in cognitive flexibility are likely mediated by increased efficiency in frontoparietal and cingulo-opercular networks following physical activity. This neural optimization results from the synergistic effects of elevated catecholamines and neurotrophic factors, which together enhance connectivity among brain regions responsible for task switching and conflict monitoring. These mechanisms align with the neural pathways described in Section 3.1 and help explain the improvements in flexible, goal-directed behavior observed after both acute and chronic exercise interventions.

### 3.5. Physical Activity and Brain Function

Brain function—defined as the coordinated activity of neural networks that support cognitive, motor, and behavioral processes—encompasses both the structural architecture and functional dynamics of the developing nervous system [61,62]. Modern neuroimaging techniques, including functional magnetic resonance imaging (fMRI), electroencephalography (EEG), and event-related potentials (ERPs), provide unprecedented opportunities to examine how physical activity influences brain activity patterns, neural efficiency, and structural connectivity in children. Brain function in childhood is characterized by dynamic developmental processes, including ongoing myelination, synaptic pruning, and the refinement of functional networks that support executive control, attention, and learning [29,63]. The protracted development of frontoparietal networks, hippocampal circuitry, and interhemispheric connectivity continues throughout childhood and adolescence, making this period particularly sensitive to environmental influences that can shape neural development [64]. Individual differences in brain function during childhood predict academic achievement, behavioral regulation, and long-term cognitive outcomes, with more efficient neural processing associated with superior performance across multiple domains [45]. Conversely, atypical patterns of brain function are linked to learning difficulties, attention problems, and challenges in executive control that can impact educational and social success [65].

The acute effects of physical activity on cognitive flexibility demonstrate small-to-moderate benefits across neurotypical pediatric populations [29]. Event-related potential studies indicate that single bouts of moderate-intensity exercise (20–30 min) produce significant increases in P3 amplitude, a marker of attentional resource allocation, during cognitive control tasks [24,26,66]. Following acute exercise, children typically exhibit larger P3 amplitudes across multiple cognitive domains, including inhibitory control, working memory, and selective attention tasks, with benefits most evident when assessment occurs within 10–20 min post-exercise [24]. Functional neuroimaging evidence demonstrates that acute moderate-intensity aerobic exercise enhances working memory-related brain activation in preadolescent children, with increased activity in bilateral parietal cortices, left hippocampus, and bilateral cerebellum during n-back task performance [22,23]. These activation changes coincide with improved behavioral performance, suggesting that acute exercise optimizes neural efficiency in memory-relevant networks [22]. Acute exercise also modulates conflict-related neurophysiological responses, with children showing reduced N2 amplitude and shorter P3 latency following moderate-intensity activities, indicating enhanced conflict monitoring and faster stimulus processing speed [24,27,28]. In preadolescents, a single ~20 min bout of moderate-intensity aerobic exercise—assessed immediately afterward—yields faster Stroop performance and increased P3 amplitude, with a concurrent reduction in conflict-sustained potential, indicating acutely enhanced attentional allocation and conflict processing [67]. Individual difference analyses reveal that children with initially lower cognitive performance show greater exercise-induced improvements in both behavioral outcomes and P3 amplitude, suggesting that acute physical activity may be particularly beneficial for those with weaker baseline cognitive control abilities [24].

The chronic effects of sustained physical activity interventions on brain function provide compelling evidence for neuroplastic adaptations following long-term exercise programs. Functional MRI results revealed that children in the physical activity intervention showed decreased activation in the right anterior prefrontal cortex during cognitive control tasks, coupled with improved behavioral performance, suggesting enhanced neural efficiency following training [20,30,31]. Event-related potential findings indicated that the intervention group maintained error-related negativity (ERN) amplitude over the 9 months, while control children showed increased ERN amplitude, with greater fitness improvements correlating with smaller ERN responses [25]. Diffusion tensor imaging showed increased fractional anisotropy and reduced radial diffusivity in the genu of the corpus callosum following the 9-month after-school physical activity program, consistent with improved white-matter organization and myelination (with no change in axial diffusivity) [68]. These microstructural changes were observed only in the intervention group, with no alterations in the wait-list controls, indicating that improvements in white-matter organization are attributable to the physical activity program rather than typical maturation over time [68]. Meta-analytic evidence from systematic reviews indicates that physical activity interventions produce significant improvements in neurophysiological functioning with moderate effect sizes, primarily reflected in enhanced P3 amplitude during cognitive control tasks [29]. Age-related analyses suggest that younger children (7–9 years) show greater responsiveness to neurophysiological interventions compared to older children (10–12 years), possibly reflecting critical periods of neural development and heightened plasticity [13]. Dose–response relationships indicate that interventions lasting 8–20 weeks with sessions of 20–40 min conducted 3–5 times per week produce optimal neurophysiological benefits [29,69]. The chronic profile reflects gradual adaptations in neural efficiency, with benefits most evident in programs that combine aerobic conditioning with cognitively engaging activities that challenge executive control networks [20,21]. Importantly, exercise-induced changes in brain function are accompanied by improvements in cognitive performance, suggesting that neuroplastic adaptations translate into meaningful behavioral benefits that support academic learning and daily cognitive demands [25,26,63].

The neurophysiological adaptations observed after acute and chronic exercise—such as greater P3 amplitude, reduced ERN, and improved frontoparietal efficiency—align closely with the mechanisms proposed in Section 3.1. Together, they illustrate how neurochemical modulation, hemodynamic facilitation, and large-scale network reorganization converge to optimize executive performance and neural efficiency in the developing brain.

**Figure 1:**
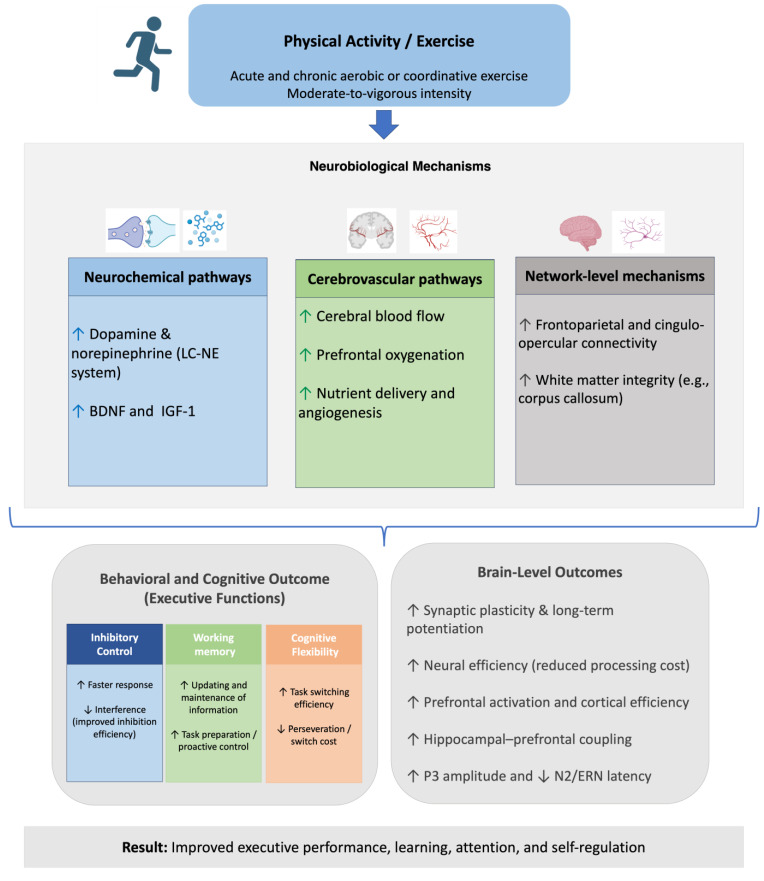
Neurobiological mechanisms of physical activity underlying executive and brain outcomes in children. Physical activity and exercise—both acute and chronic, aerobic or coordinative, and performed at moderate-to-vigorous intensity—activate multiple neurobiological pathways. These include neurochemical mechanisms (↑ dopamine and norepinephrine via the LC–NE system; ↑ BDNF and IGF-1), cerebrovascular adaptations (↑ cerebral blood flow, ↑ prefrontal oxygenation, ↑ nutrient delivery and angiogenesis), and network-level changes (↑ frontoparietal and cingulo-opercular connectivity; ↑ white-matter integrity, e.g., corpus callosum). These processes support improvements across executive-function domains: inhibitory control (↑ faster responses; ↓ interference), working memory (↑ updating and maintenance of information; ↑ task preparation/proactive control), and cognitive flexibility (↑ task-switching efficiency; ↓ perseveration/switch cost). At the brain level, physical activity enhances synaptic plasticity and long-term potentiation, neural efficiency (reduced processing cost), prefrontal activation, hippocampal–prefrontal coupling, and electrophysiological markers (↑ P3 amplitude and ↓ N2/ERN latency). Ultimately, these adaptations contribute to better learning, attention, self-regulation, and academic performance in children. Upward arrows (↑) represent increases or enhancements, whereas downward arrows (↓) reflect reductions associated with beneficial effects (e.g., decreased interference, reduced processing cost, or shorter N2/ERN latencies).

Collectively, these findings converge on a multi-level account linking exercise-induced neurochemical, cerebrovascular, and network-level changes to executive and brain outcomes in children. Figure 1 synthesizes the Neurobiological mechanisms of physical activity underlying executive and brain outcomes in children.

### 3.6. Physical Activity in Children with Neurodevelopmental Disorders

Neurodevelopmental disorders—including ADHD, autism spectrum disorder (ASD), and learning disabilities—are characterized by persistent cognitive and behavioral difficulties, often linked to deficits in executive functions [70,71,72]. The burden of neurodevelopmental disorders is substantial. In a nationally representative U.S. sample of children and adolescents (ages 3–17), the weighted prevalence was 8.5% for ADHD, 2.9% for autism spectrum disorder, 1.4% for intellectual disability, and 6.4% for learning disability. Rates were higher in boys and among youth with co-occurring anxiety or depression, lower family income, rented housing, a history of bullying, or household mental illness—factors that compound cognitive and psychosocial impairment and adversely affect quality of life, long-term prognosis, and treatment needs [73]. In the absence of adequate intervention, symptoms often persist from childhood into adolescence and even adulthood, underscoring the urgent need to identify effective, evidence-based interventions [74].

Multiple neurobiological pathways plausibly explain how physical activity can enhance executive functions in children with neurodevelopmental disorders. In ADHD, dysfunction within specific circuits—including the frontoparietal network and the dorsal and ventral attention networks—and network hyperactivation are linked to executive deficits [75]. Physical-activity interventions can engage brain regions subserving executive control and increase functional connectivity among large-scale networks, thereby supporting executive function in children with ADHD [76,77,78]. ADHD is also associated with imbalances in catecholaminergic neurotransmission [79,80]. Exercise has been shown to increase the release of catecholamines such as dopamine and norepinephrine, elevating cortical arousal and, in turn, promoting the development of executive functions in children with ADHD [76,81].

In autism spectrum disorder, evidence suggests that exercise interventions can modify synaptic function and promote behavioral improvements by modulating the structural plasticity of synapses and dendritic spines [82]. Abnormal alterations in dendritic-spine density may disrupt specific neural circuits, and exercise may counter these patterns through regulation of neurotrophic factors [83]. Physical activity also modulates the hypothalamic–pituitary–adrenal (HPA) axis, effectively reducing stress hormones such as cortisol and alleviating neural stress load [84]. Lower stress levels contribute to greater emotional stability and reduce cognitive load, enabling children with neurodevelopmental disorders to focus more effectively on information processing.

A single session of physical activity can produce immediate benefits in executive functions and brain activity in children with neurodevelopmental disorders. In children with ADHD and other neurodevelopmental disorders, including ASD, single 15–30 min bouts of moderate-to-vigorous physical activity—whether aerobic or cognitively engaging (e.g., exergaming or coordinative exercise)—have been shown to produce reliable improvements in inhibitory control, cognitive flexibility, and task-switching performance, with accuracy generally maintained, while effects on working memory may be less consistent [85,86]. In parallel, a meta-analysis reports increases in P3 (and, in some cases, N2) amplitude, shorter latencies, and greater prefrontal oxygenation during cognitive performance, consistent with more efficient allocation of attentional resources [86].

Chronic physical activity interventions have been shown to produce significant improvements in overall executive functions and their subdomains—inhibitory control, cognitive flexibility, working memory, and higher-level functions—primarily in children and adolescents with ADHD, and to a lesser extent in those with ASD and other neurodevelopmental disorders [85,86,87,88]. The magnitude of these effects is moderated by multiple factors, including session duration, number of weeks, total number of sessions, and total intervention duration [86]. Interactions between session characteristics, physical activity type, frequency, and executive function subdomains indicate that interventions lasting 45–70 min per session, conducted at least three times per week over multiple weeks, are particularly effective [86]. While inhibitory control appears to be the most sensitive domain and often shows initial improvements, these gains may facilitate subsequent enhancements in cognitive flexibility and higher-level functions, eventually supporting working memory development [86]. Longer sessions exceeding 70 min can also elicit benefits, particularly when a combination of aerobic and cognitively engaging exercises is employed, which may help prevent mental fatigue and maintain cognitive engagement [86]. These findings suggest that regular and repeated practice over time is crucial for maximizing the cognitive benefits of physical activity in this population, and that chronic interventions may induce more robust and sustained improvements than single, acute sessions.

The neurobiological pathways outlined in Section 3.1—particularly catecholaminergic modulation and BDNF-driven neuroplasticity—play a crucial role in not only transiently enhancing cortical arousal but also promoting long-term functional network reorganization. These adaptations support sustained improvements in executive functions and cognitive control in children with neurodevelopmental disorders, underscoring the importance of regular, repeated physical activity as part of therapeutic strategies.

## 4. Conclusions

The evidence reviewed consistently indicates that physical activity is a significant factor in the development of executive and brain functions in childhood. Both acutely and chronically, exercise promotes improvements in inhibition, working memory, and cognitive flexibility, supported by neurobiological mechanisms that include neurotransmitter modulation, increased neurotrophic factors, greater cerebral oxygenation, and structural and functional adaptations in cortical and subcortical networks. These transformations suggest that the benefits of physical activity are not only short-term but can be consolidated through sustained practice, contributing to greater cognitive and brain efficiency during critical stages of development.

In the educational field, the findings support the promotion of active lifestyles at school, given their association with better academic performance and more adaptive classroom behavior. Interventions that incorporate active play, sports, and coordinative activities not only promote physical health but also serve as pedagogical tools with the potential to improve attention, planning, and problem-solving in students.

From a clinical perspective, physical activity appears to be a promising complementary strategy for children with neurodevelopmental disorders, especially those with ADHD and, to a lesser extent, ASD. The benefits observed in inhibition, flexibility, self-regulation, and brain functioning suggest that well-designed exercise programs can be integrated as non-pharmacological support in therapeutic plans, helping to reduce impulsivity and improve sustained attention.

Future research should prioritize comparative designs including both neurotypical children and those with neurodevelopmental disorders—particularly ADHD and ASD—to better delineate the shared and distinct neurobiological pathways through which exercise influences executive functioning.

Most of the studies included in this review presented methodological limitations, such as small and heterogeneous samples, short intervention durations, and variability in the cognitive tasks and exercise protocols applied. These factors restrict comparability across studies and limit the strength of causal inferences regarding the effects of physical activity on executive and brain functions. Nevertheless, important gaps remain in the literature. The heterogeneity of protocols, the scarcity of longitudinal studies, and the limitations in generalizing the results highlight the need for more robust and standardized research to determine with greater precision the dose, type, and intensity of physical activity most suitable for each population. Furthermore, studies conducted in school and community settings are needed to assess the real-world impact of these interventions on children’s daily lives.

Overall, physical activity emerges as a key tool to promote the development of executive and brain functions in childhood. Encouraging regular practice from early ages represents not only an investment in physical health but also in the academic, socioemotional, and neurological potential of future generations, with direct implications for both education and public health.

## Data Availability

Not applicable. This study is based on previously published literature, and no new data were generated or analyzed.

## Abbreviations

The following abbreviations are used in this manuscript:

ADHD	Attention-Deficit/Hyperactivity Disorder
BDNF	Brain-Derived Neurotrophic Factor
EEG	Electroencephalography
ERP	Event-Related Potential
fMRI	Functional Magnetic Resonance Imaging
RCT	Randomized Controlled Trial
ASD	Autism Spectrum Disorder
